# Theoretical Design of Optimal Molecular Qudits for Quantum Error Correction

**DOI:** 10.1021/acs.jpclett.2c01602

**Published:** 2022-07-11

**Authors:** A. Chiesa, F. Petiziol, M. Chizzini, P. Santini, S. Carretta

**Affiliations:** † Università di Parma, Dipartimento di Scienze Matematiche, Fisiche e Informatiche, I-43124 Parma, Italy; ‡ Gruppo Collegato di Parma, INFN−Sezione di Milano-Bicocca, 43124 Parma, Italy; ¶ Institut für Theoretische Physik, Technische Universität Berlin, Hardenbergstr. 36, 10623 Berlin, Germany; § UdR Parma, INSTM, I-43124 Parma, Italy

## Abstract

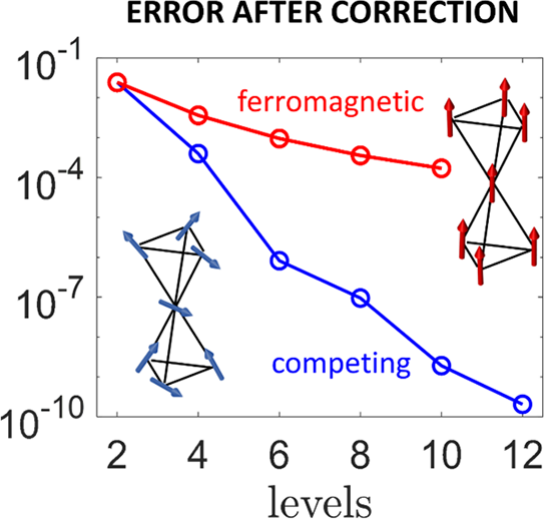

We pinpoint the key ingredients ruling decoherence in multispin clusters, and we engineer the system Hamiltonian to design optimal molecules embedding quantum error correction. These are antiferromagnetically coupled systems with competing exchange interactions, characterized by many low-energy states in which decoherence is dramatically suppressed and does not increase with the system size. This feature allows us to derive optimized code words, enhancing the power of the quantum error correction code by orders of magnitude. We demonstrate this by a complete simulation of the system dynamics, including the effect of decoherence driven by a nuclear spin bath and the full sequence of pulses to implement error correction and logical gates between protected states.

Quantum computers promise to outclass classical digital devices in the solution of currently intractable problems. However, none of the existing technologies^1−10^ can suppress errors on each computational qubit to the level required to achieve a real quantum advantage. The only way to get around this hurdle is by replacing two-level qubits with more complex logical units, supporting quantum-error correction (QEC). Each of these units is usually represented by a large collection of qubits,^11,12^ at least 10^3^–10^4^ to get, for example, a likely success in factoring a 2000-bit number.^13^ Even for the most advanced platforms, this makes the actual manipulation of the resulting register practically unfeasible and hence still represents an almost prohibitive goal.

Molecular spin systems offer a new alternative perspective, which can overcome major limitations of qubit-based approaches. In particular, they are typically characterized by many electronic and nuclear spin states (i.e., a *qudit* structure), which can be coherently manipulated through microwave or radio frequency pulses.^14−20^ We have recently shown that these qudits can be exploited to embed QEC within a single object, thus greatly simplifying its actual implementation.^21,22^ The 2*S* + 1 states of a spin *S* ion (for which several examples exist^23−29^) provide the chemically simplest implementation, which already ensures a gain in the lifetime of a quantum memory.^21,22^ By increasing *S*, and hence the number of levels in the encoding, one could in principle increase the correcting capacity of the code.

However, the dramatic growth of decoherence with *S* yields only a limited gain in actually performing quantum error correction. Moreover, quantum gates between logical states encoded in spin *S* qudits are not allowed. Instead, to implement complex algorithms, logical units must display errors not increasing significantly with the system size and must support gates between encoded states.

Here we show that both these challenging tasks can be achieved by fully unleashing the chemical tunability of our molecular hardware, which constitutes its fundamental advantage but was not exploited to date. In particular, we theoretically design optimal molecules showing a large number of low-energy states, for which decoherence is strongly suppressed and does not grow with the system size. This allows us to increase the number of levels in the encoding without being limited by the corresponding loss of coherence. As a result, the correcting power of the code is largely enhanced, by orders of magnitude compared to the case of a spin *S* system. The optimal units are represented by multispin molecules with antiferromagnetic competing exchange interactions,^30−34^ leading to several magnetically similar multiplets at low energy. As a consequence, superpositions of all the states belonging to these multiplets are substantially protected from decoherence in a way that does not worsen by adding levels.

We demonstrate this by considering a 7-spin molecule in a bath of nuclear spins, driving decoherence at low temperature. We numerically compute the resulting effect of dephasing on the lowest energy levels, and we derive code words exploiting superpositions of these levels. We then compare the performance of the QEC code for the same molecular structure with competing versus ferromagnetic exchange interactions (producing a ground spin *S* multiplet), finding an impressive gain for the former. Finally, we exploit another peculiar feature of the designed molecule, namely, the possibility to induce direct transitions between all the selected energy levels, to actually implement quantum error correction and quantum gates between encoded states.

*Design of Molecular Nanomagnets with Suppressed Decoherence*. To design optimal molecular systems, we first pinpoint the crucial ingredients related to the spin structure of the eigenstates driving decoherence and then identify the requirements to keep them under control. The dominant source of decoherence in molecular nanomagnets at low temperature is the hyperfine coupling of the system spins with the surrounding nuclear spins, while phonon-mediated processes are practically negligible.^22,23^ Starting from the microscopic system-bath Hamiltonian and from the system eigenstates, we derive in the Supporting Information a master equation describing the dynamics of the system in the secular and Born–Markov approximations. This yields a decay of the system coherences ρ_*μν*_(*t*) = exp(− γ_*μν*_*t*)ρ_*μν*_(0) with decay rates
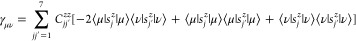
1Here, ρ is the density matrix, |μ⟩ and |ν⟩ are system eigenstates, and the coefficients  contain sums over products of dipolar couplings between local system and bath spin operators.^49^ Although neglecting the details of the spin-bath dynamics, this approach captures the dramatic impact of the spin structure of the eigenstates on the dephasing process, the key ingredient for the design of optimal molecules. Indeed, as demonstrated in the Supporting Information, the detailed description of the bath dynamics does not qualitatively alter our main conclusions.^50^

The key factors ruling decoherence in eq 1 are weighted differences between expectation values of local spin operators on different eigenstates. In order to suppress decoherence and in particular to avoid its growth when additional states are considered, the values of  and their variation within the examined set of eigenstates must be as small as possible. This suggests as ideal candidates antiferromagnetically coupled systems with competing exchange interactions (i.e., close to spin frustration), which are expected to display several “magnetically similar” low-energy multiplets, i.e., characterized by similar and small values of .

Quasi-frustration is typically associated with the presence of triangular units with antiferromagnetic coupling in the molecule. For example, a single triangle of *s*_*i*_ = 1/2 spins may provide two low-energy multiplets with small total spin *S* = 1/2. This is not enough for the present study, where we want to compare the performance of the QEC protocol across a broad range of qudit sizes. Thus, we consider a bigger molecule whose structure remains as simple as possible but which can accommodate a larger number of suitable low-energy multiplets: two corner-sharing tetrahedra of antiferromagnetically coupled spins (Figure 1a), a structure analogous to that of the Ni_7_ cluster.^30^ A simple choice yielding a large number of low-lying multiplets is six spins *s*_*i*_ = 1/2 (*i* = 1, ..., 6) and a spin *s*_7_ = 3/2 (red spheres and arrows) at the shared corner. The system is described by the following spin Hamiltonian:
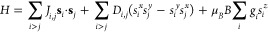
2where the first term represents the (leading) isotropic exchange interaction between different ions connected by red lines in Figure 1a with coupling strength *J*_*i*,*j*_; the second is the axial Dzyaloshinskii–Moriya interaction (DMI), parametrized by *D*_*i*,*j*_; and the latter is the Zeeman coupling of each ion with an external magnetic field parallel to *z*. For simplicity, we have assumed isotropic *g*_*i*_ factors and only axial terms in Hamiltonian 2. In the case of isosceles triangles at top and bottom with all their vertices coupled with the same strength to the center, the energies of the isotropic exchange multiplets can be analytically computed (see the Supporting Information). This allows us to identify a proper regime of parameters to get several low-energy multiplets with minimum total spin *S* = 1/2. In particular, by choosing positive (antiferromagnetic) values for all *J*_*i*,*j*_ and the intratriangles coupling substantially larger than the coupling with the center, the spectrum displays eight doublets significantly separated from the first excited *S* = 3/2. This leads to a situation close to spin frustration,^34^ with the degeneracy of the ground state removed by using slightly different *J*_*i*,*j*_, as listed in the caption of Figure 1. This is the typical situation found in real molecules, which are usually not completely regular,^30−33,35−41^ and is exactly our target, because such a symmetry breaking makes all transitions addressable in principle by resonant pulses (see the Supporting Information). Finally, DMI is exploited to couple to first order all different multiplets, thus providing matrix elements for direct transitions. We fix *D*_*i*,*j*_ = *J*_*i*,*j*_/10, a reasonable assumption for, e.g., Cu^2+^ spin 1/2 ions with *g*_*i*_ ≈ 2.1–2.3; see ref (42).

**Figure 1:**
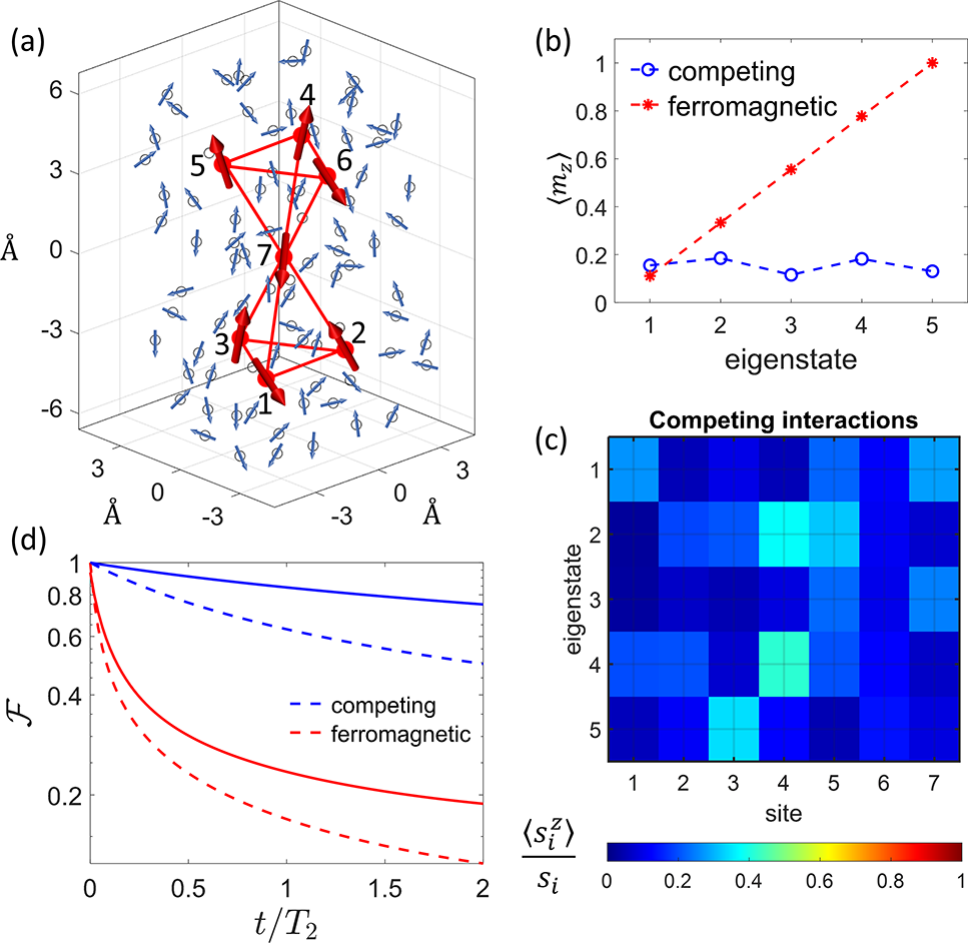
Suppression of decoherence in a multispin molecule with competing interactions. (a) Hypothetical multispin cluster consisting of six *s*_*i*_ = 1/2 and a spin 3/2 ion in the center (such as Cu^2+^ and Cr^3+^, red arrows), arranged in a double-tetrahedron structure similar to Ni_7_.^30^ Calculations are performed by assuming *J*_1,2_ = 1.14 meV, *J*_1,3_ = *J*_2,3_ = 1.15 meV, *J*_4,5_ = 1.13 meV, *J*_4,6_ = *J*_5,6_ = 1.10 meV, *J*_1,7_ = 0.82 meV, *J*_2,7_ = 0.85 meV, *J*_3,7_ = 0.87 meV, *J*_4,7_ = 0.83 meV, *J*_5,7_ = 0.81 meV, *J*_6,7_ = 0.90 meV, and *D*_*i*,*j*_ = *J*_*i*,*j*_/10. The system is surrounded by a random distribution of nuclear spins 1/2 (blue arrows) with minimal distance 2 Å centered around each ion, ruling decoherence. (b) Comparison between the average expectation values of local spin operators  in the case of competing (blue) vs ferromagnetic (red) exchange interactions in the molecular structure shown in panel a, for different sites and eigenstates (one per each of the lowest 5 Kramers doublets). (c) Distribution of expectation values of local spin operators  on different sites and eigenstates of the cluster (a) with competing interactions. The much larger variation of ⟨*m*_*z*_⟩ within the *S* = 9/2 ground multiplet in panel b is reflected (panel d) by the impressive reduction of the fidelity for a state prepared in an initial generic uniform superposition (dashed lines) or in the encoded state  (solid). Here , where |ψ_0_⟩ is the initial state and ρ is the system density matrix subject to decoherence for a time *t*.

To quantitatively show that this system with *competing* interactions (**C**) fulfils the aforementioned requirements, we consider its lowest eigenstates and compute expectation values of local spin operators. In particular, we compare it with an iso-structural molecule in which the sign of all *J*_*i*,*j*_ is reversed, leading to an *S* = 9/2 ground multiplet (referred to hereafter as *ferromagnetic*, **F**). The latter perfectly matches the spin *S* cases examined previously,^21,22,43^ but in the same bath of **C**. The average local spin moment over different sites  is reported in Figure 1b for different eigenstates (a single one per Kramers pair). In the case of **F** and within the *S* = 9/2 ground multiplet, |μ⟩ ≡|*m*⟩, where |*m*⟩ are the common eigenstates of *H* and *S*_*z*_ = ∑_*i*_*s*_*i*_^*z*^, *S*_*z*_|*m*⟩ = *m*|*m*⟩. Hence, one can easily find . This results in a strong variation of ⟨*m*_*z*_⟩ among different eigenstates (red symbols), in contrast to the case of **C**, where ⟨*m*_*z*_⟩ is very weakly dependent on the state. This is also evident from panel c, where we report the detailed distribution of  in **C** over different sites, showing again small values with a rather uniform distribution on the eigenstates. The different dependence of ⟨*m*_*z*_⟩ on the examined eigenstate translates into a very different coherence decay. This is highlighted in Figure 1d, where we compare the fidelity decay of different superpositions of the 10 lowest states for **F** and **C**. The larger the variation of ⟨*m*_*z*_⟩ in panel b, the faster the decay. This can be easily understood for **F**, in which case γ_*mm*′_ = (*m* – *m*′)^2^/*T*_2_, where we have introduced an effective coherence rate , containing information on the bath and common to both **F** and **C**. All in all, Figure 1 provides insight into the crucial ingredients driving decoherence and illustrates our recipe to suppress it and to avoid its growth with the number of states by properly engineering the couplings in Hamiltonian 2.

*Deriving Optimal Code Words*. Building on the above model of decoherence, we show how to derive a hardware-efficient quantum error correction code capable of substantially suppressing errors. This is achieved in two steps. First, we focus on the *d* lowest levels of the system and we decompose ρ at time *t* through a set of operators *E*_*k*_ in this subspace:
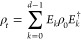
3Starting from the solution of the Lindblad equation , the *d* independent diagonal *E*_*k*_ operators are derived through state tomography (see the Supporting Information and ref (44)). The second step consists of identifying logical states (*code words*) |0_*L*_⟩ and |1_*L*_⟩, i.e., proper superpositions of the system eigenstates satisfying Knill–Laflamme conditions^45^ on the subset of leading *d*/2 error operators {*E*_*k*_}:^51^
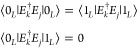
4The second condition is guaranteed by choosing different |μ⟩ states to define |0_*L*_⟩ and |1_*L*_⟩, being all *E*_*k*_ diagonal. The first one leads to a linear system of equations for the squared coefficients of the logical states on the |μ⟩ basis of states, for which at least one solution can be found, either by matrix pseudoinversion or by numerical optimization (see the Supporting Information). Figure 2a shows as a bar plot the absolute value of the components on each eigenstate of the code words (derived at *t*/*T*_2_ = 0.05) for **C**.

**Figure 2:**
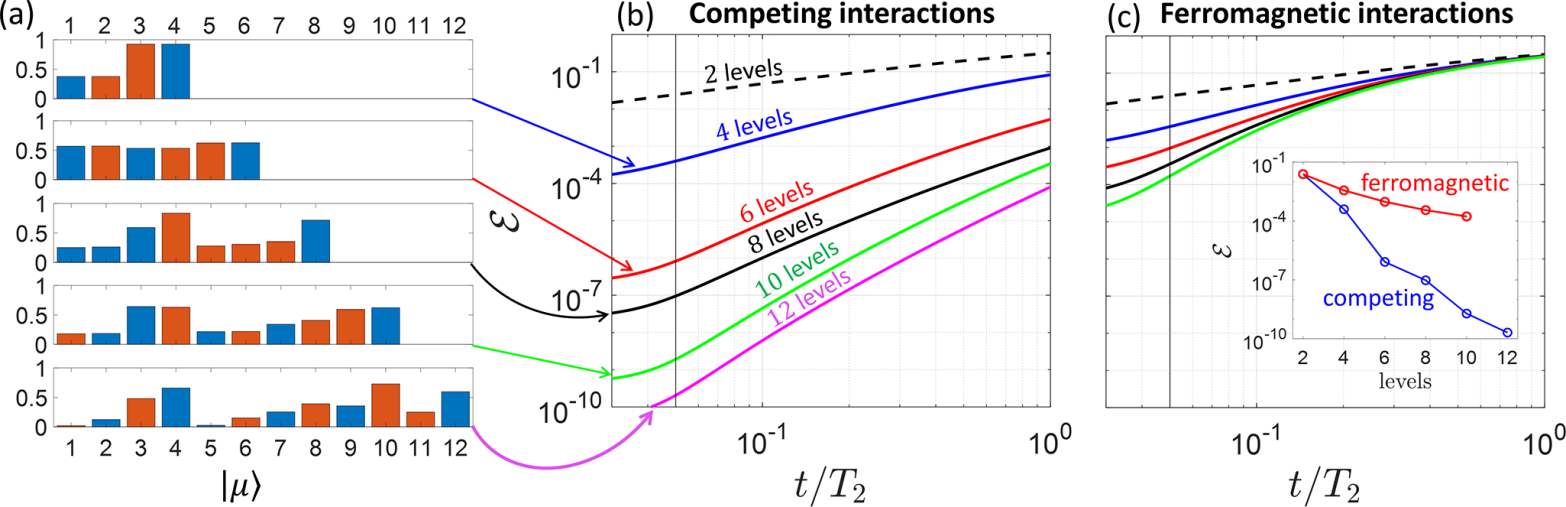
Quantum error correction using a properly designed molecular qudit. (a) Absolute value of the component of each code word on the eigenstates of **C**, labeled in the horizontal axis by a number from 1 to 12. Blue (orange) bars refer to |0_*L*_⟩ (|1_*L*_⟩). (b and c) Error  after a memory time *t* in the molecular system of Figure 1a with competing (b) vs ferromagnetic (c) exchange interactions, exploiting an increasing number of levels for the encoding and starting from the error-prone state . The code words are derived at *t*/*T*_2_ = 0.05 (vertical line). The comparison shows an impressive gain in panel b compared to panel c, increasing with the number of levels up to 5 orders of magnitude (inset of panel c) at *t*/*T*_2_ = 0.05.

To test the performance of the optimized code words, we perform an ideal QEC cycle applied after memory time *t*. This is done by starting from a generic superposition state |ψ_0_⟩ = α|0_*L*_⟩ + β|1_*L*_⟩ and letting the system evolve freely (only subject to decoherence) for time *t*. Then, we check if each of the *E*_*k*_ errors has occurred by projecting the state of the system into one of the *d*/2 error words , i.e., an orthonormal set of states spanning the same Hilbert space as {*E*_*k*_|0_*L*_⟩, *E*_*k*_|1_*L*_⟩}.^21^ Thanks to the structure of the code words which satisfy eq 4, the resulting state  still preserves the initially stored information. Hence, we can finally apply the corresponding recovery operation  to restore the logical state. Results of this procedure are reported in Figure 2b,c. As expected, the error  is dramatically lower in **C** (b) compared to **F** (c). This is also evidenced in the inset, where  is shown as a function of the number of levels used for the encoding. In moving from **F** to **C**,  is suppressed up to 5 orders of magnitude with 10 levels (the maximum number for a *S* = 9/2 multiplet). In principle, the error could be further reduced by deriving code words at shorter times and/or including a larger number of levels in the encoding.

*Benchmarking Quantum Error Correction*. The actual implementation of QEC requires a further step: a clear scheme to identify, measure, and correct errors. Here we present efficient strategies to achieve this, exploiting the peculiar structure of the eigenstates of **C**. The simplest one is based on measuring directly the state of the qudit, which can be done on the basis of the system eigenstates |μ⟩. However, the system is in general in a superposition of different eigenstates and of different error words. Hence, in order to perform a measurement that distinguishes different errors we need to map each error word  into a specific pair of system eigenstates α|μ_0_⟩ + β|μ_1_⟩. Such a mapping can be directly achieved by a sequence of parallel pulses thanks to the connectivity between the energy levels, which enables direct transitions among all of them. Then, simultaneous measurement of the pair of levels |*μ_l_*⟩ (*l* = 0,1) allows one to identify the error word without collapsing the encoded state^21,22^ and to apply the corresponding recovery.

This procedure hides, however, a potential drawback: by mapping each error word into a specific eigenstate (*decoding*), we leave the protected state for some time. To avoid this, we propose an alternative error detection scheme, in which measurements are performed on the eigenstates of an ancillary *d*/2-level qudit (e.g., a single electronic or nuclear spin or another molecule), which flag each of the *d*/2 errors *E*_*k*_. The idea is to apply a sequence of pulses, detailed in the circuit of Figure 3a and in the Supporting Information, entangling system and ancilla in such a way that in the output superposition each state of the ancilla is in one-to-one correspondence with one of the error words (and hence, in practice, with one of the errors *E*_*k*_). Then, by measuring the ancilla in its eigenbasis we project the state of the logical qudit into a specific error word and we can apply the corresponding recovery R_*k*_.

**Figure 3:**
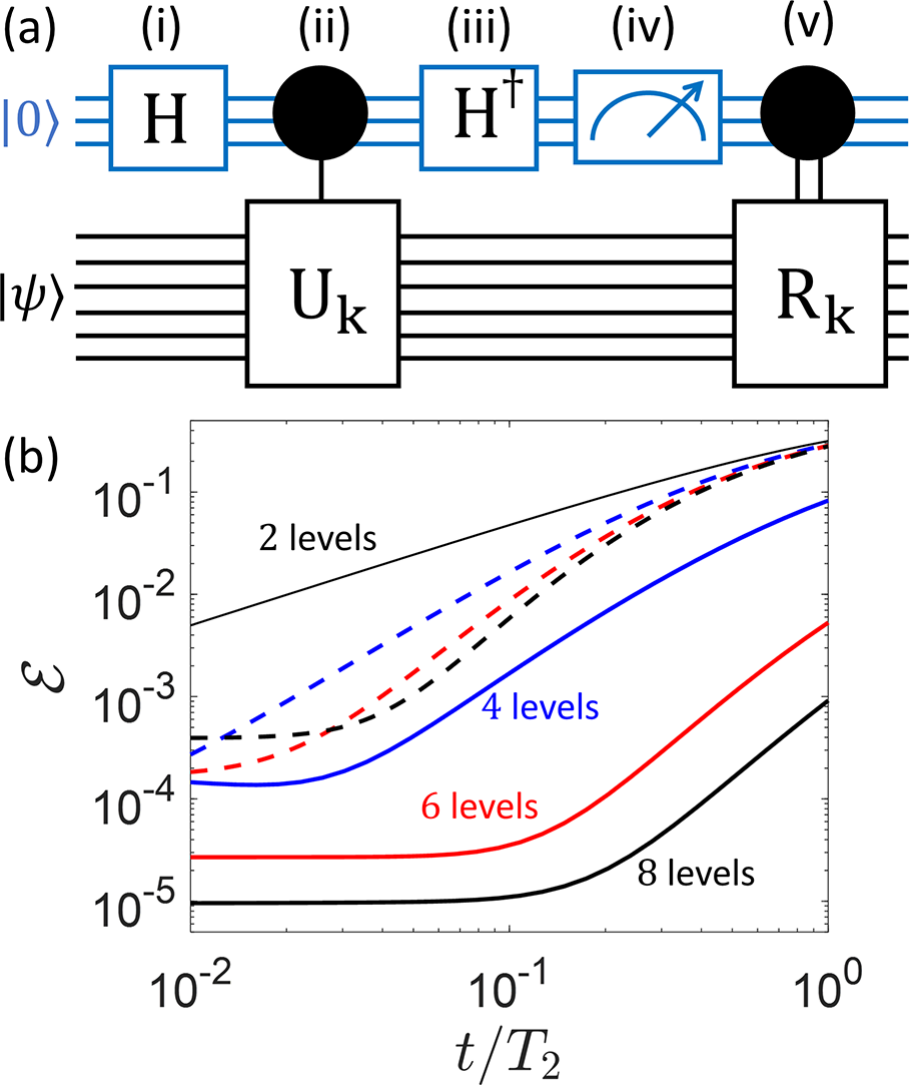
(a) Quantum circuit to detect error *E*_*k*_ on a *d* level qudit (bottom black lines), exploiting a *d*/2 level qudit ancilla (top blue lines): Starting with the ancilla in the ground state |0⟩ and the logical qudit in |ψ_0_⟩ = α|0_*L*_⟩ + β|1_*L*_⟩, we first (i) prepare the ancilla in a uniform superposition of its *d*/2 levels (through a generalized Hadamard gate H). Then, (ii) we implement a different unitary evolution U_*k*_ (one for each error *E*_*k*_) on the logical qudit, conditioned by each of the |*k*⟩ eigenstates of the ancilla. Next, (iii) we apply the inverse of transformation (i) to the ancilla and (iv) measure its state, projecting the system into a specific error word. Finally, a (v) specific recovery R_*k*_ is applied, depending on the measurement outcome. (b) Resulting error after a memory time *t*/*T*_2_, using the whole pulse sequence to detect and correct errors and exploiting an increasing number of levels for the encoding. Here, *T*_2_ = 10^5^τ, with τ being the largest time required to implement an elementary π pulse. Continuous (dashed) lines refer to **C** (**F**).

The sequence of operations outlined in Figure 3a requires conditional ancilla–qudit U_*k*_ gates. Their implementation by microwave pulses requires distinguishing excitations of **C**, depending on each state of the ancilla. This, in turn, implies a sufficient qudit–ancilla coupling to spectroscopically resolve all of them. Then, each unitary U_*k*_ is implemented by two sets of simultaneous pulses while always keeping the system encoded, as detailed in the Supporting Information. The state of the ancilla can be finally read out by coupling it to a superconducting resonator and measuring its frequency shift.^46^ Remarkably, thanks to the full connectivity between energy levels ensured by the DMI in Hamiltonian 2, the length of the sequence does not increase with the number of states.

This, combined with the suppression of decoherence, yields a much better performance than **F**, as can be seen by comparing solid and dashed lines in Figure 3b. In particular, the error at short *t*/*T*_2_ increases in **F** by increasing the number of levels, due to both the growth of decoherence and of the number of pulses to implement the correction.^21^ In contrast, **C** shows a reduction of  by increasing *d*.^52^ The reported numerical simulations include the whole pulse sequence and the effect of pure dephasing on the qudit, but we assume perfect pulses (with no leakage to neighboring levels). Indeed, pulse imperfections strongly depend on specific details of the system Hamiltonian and of the experimental apparatus and could be significantly suppressed by optimal quantum control techniques,^47^ which is beyond the scope of the present work. Using the spin Hamiltonian parameters listed above (chosen to well resolve all the transitions), electron paramagnetic resonance frequencies up to the W-band are needed. However, we stress that by using optimal quantum control to shape pulses these frequencies could be reduced (e.g., by an overall reduction of the coupling in Hamiltonian 2) without significantly affecting gate fidelities. Targeting molecules with smaller couplings would also be advantageous to decrease the rate of spin–lattice processes, in case phonon-induced relaxation turned out to be disturbing. Yet, such an effect is expected to be minor. For example, for the frustrated Cu_3_ triangle studied in ref (48), *T*_1_ values as large as hundreds of microseconds were observed, in spite of gaps much bigger than in our case.

*Protected Quantum Gates*. The total connectivity between the energy levels involved in the encoding also allows us to design a scheme to implement generic *R*_α_(ϑ) logical gates between encoded states. We focus here on the more demanding planar rotations *R*_*x*,*y*_, because diagonal one- and two-qubit gates can be implemented more easily.^43^ These are obtained by inducing simultaneous transitions between each component of |0_*L*_⟩ and those of |1_*L*_⟩, without decoding (i.e., mapping each logical state into a single level). In contrast, implementing the same gate on **F** would require a long sequence of pulses, practically equivalent to decoding, applying the rotation, and encoding again. As a result, the performance of **C** is much better already for a *d* = 4 qudit, as highlighted in Figure 4. Here we report numerical simulations of a sequence of *R*_*x*_(π) rotations, followed by a short memory time (as it would happen in a complex algorithm) and by a QEC cycle. While the final error in **F** (red crosses) is always larger than that on a not protected spin 1/2 qubit (dashed line), **C** reveals a significant advantage (blue symbols), even with application of the full pulse sequence (circles). This translates into a gain (panel b) compared to the spin 1/2 approaching 4 after implementation of 30 gates, which is remarkable having exploited the qudit with minimum protection, namely, *d* = 4.

**Figure 4:**
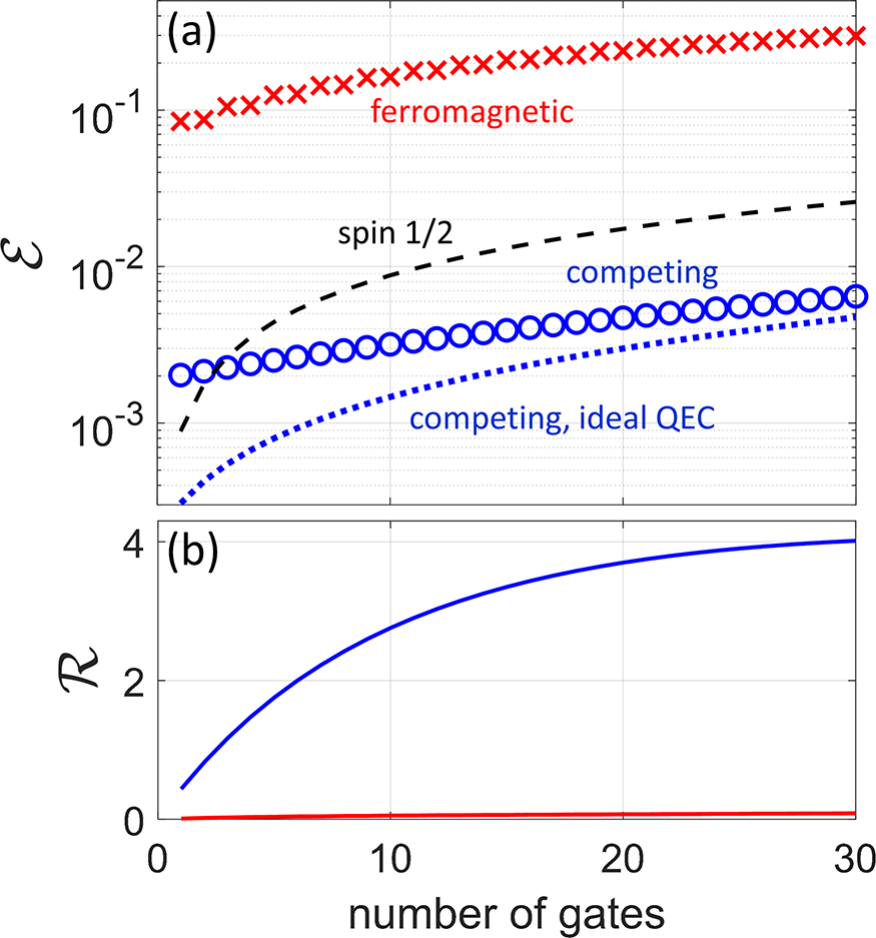
Protected quantum gates on a four-level qudit. Error  (a) and gain  compared to an uncorrected spin 1/2 (i.e. ratio between errors in the un-corrected and corrected cases) (b) in the implementation of a sequence of *R*_*x*_(π) rotations of the logical qubit of duration τ, each one followed by a memory time 2τ and by a QEC cycle. Blue circles (red crosses) refer to **C** (**F**). In the former case, the connectivity between the system eigenstates allows us to perform generic *R*_*x*_(ϑ) rotations much faster and without decoding, resulting in a much better performance. Blue dots are the result of a noisy gate, followed by an ideal QEC cycle, while the dashed line is the error for an uncorrected qubit, subject to the same gate and memory time. In the simulations, leakage is neglected and we assume *T*_2_ = 10^3^τ.

In summary, we have theoretically designed molecular nanomagnets showing a striking performance as qubits with embedded quantum error correction. This is achieved through an insight into the role of the spin structure of the eigenstates on the mechanisms ruling decoherence, which allowed us to identify as optimal units multispin molecules with competing exchange interactions. Indeed, these systems are characterized by several low-energy multiplets where decoherence is strongly suppressed and does not grow with the number of levels in the encoding. In this respect, one could also engineer molecular systems embedding an increasingly large number of long-coherence states at low energy.^33,34^ In addition, these levels are directly connected by microwave transitions. These two features allow us to derive protected logical states and to design efficient pulse sequences to actually implement quantum error correction and quantum gates.

## Supporting Information Available

Details on the energy spectrum of the examined systems, derivation of the master equation, state tomography and determination of the code words, pulse sequence to implement quantum error correction and logical gates (PDF)
