# Supplementary material for: Theoretical Design of Optimal Molecular Qudits for Quantum Error Correction
Source: J Phys Chem Lett. 2022 Jul 11;13(28):6468–74. doi: 10.1021/acs.jpclett.2c01602 (PMC9310095; doi:10.1021/acs.jpclett.2c01602)
Supplement: Supplementary file 1 — jz2c01602_si_001.pdf [file jz2c01602_si_001.pdf]

Supporting Information: Theoretical Design of Optimal Molecular Qudits for Quantum Error Correction

A. Chiesa,[*,†] F. Petiziol,[§] M. Chizzini,[†] P. Santini,[†] and S. Carretta[*,†]

†*Università di Parma, Dipartimento di Scienze Matematiche, Fisiche e Informatiche, I-43124 Parma, Italy*

‡*UdR Parma, INSTM, I-43124 Parma, Italy*

¶*INFN–Sezione di Milano-Bicocca, gruppo collegato di Parma, 43124 Parma, Italy*

§*Institut für Theoretische Physik, Technische Universität Berlin, Hardenbergstraße 36, 10623 Berlin, Germany*

E-mail: alessandro.chiesa@unipr.it; stefano.carretta@unipr.it

Energies of the exchange multiplets

By considering two isosceles triangles at the bottom and top of Fig. 1-(a) in the main text with all the vertexes coupled with the same interaction strength to the centre, the isotropic exchange Hamiltonian is diagonal on the total spin basis $|S_{12}\ S_{123}\ S_{45}\ S_{456}\ S_{1-6}\ S\rangle$. Here $S_{12} = s_1 + s_2$, $S_{123} = S_{12} + s_3$, $S_{45} = s_4 + s_5$, $S_{456} = S_{45} + s_6$, $S_{1-6} = S_{123} + S_{456}$, $S = S_{1-6} + s_7$.

The energy of the exchange multiplets results:

$$\begin{aligned} E(\{S_\alpha\}) &= (J_{1,2} - J_{1,3})\ S_{12}\ (S_{12}+1) \\ &+ J_{1,3}\ S_{123}\ (S_{123}+1) \\ &+ (J_{4,5} - J_{4,6})\ S_{45}\ (S_{45}+1) \\ &+ J_{4,6}\ S_{456}\ (S_{456}+1) \\ &+ J_{1,7}\ [S(S+1) - S_{1-6}\ (S_{1-6}+1)], \end{aligned}$$

where $J_{1,3} = J_{2,3}$, $J_{4,6} = J_{5,6}$ and $J_{1,7} = J_{2,7} = J_{3,7} = J_{4,7} = J_{5,7} = J_{6,7}$. From this expression it is easy to explore the parameters space and to identify conditions in which several $S = 1/2$ multiplets lie in the lowest part of the spectrum. In particular, the crucial parameter is the ratio between intra-triangles ($J_{i,j}$, $i, j = 1, ..., 6$, J hereafter) and the triangles to centre ($J_{i,7} \equiv J'$) couplings. Fig. S1-(a) reports the energy of the $S = 3/2$ (black lines) and $S = 1/2$ (red) exchange multiplets as a function of J/J'. For $J/J' \gtrsim 1.2$ we find eight doublets below the first $S = 3/2$.

Fig. S1-(b) also shows the lowest energy-level diagram as a function of the external field of the full Hamiltonian (1) of the main text including anisotropy. The latter is crucial to enable electron-paramagnetic resonance (EPR) transitions between different multiplets by means of microwave pulses. To simplify the picture, we have chosen a fully axial Hamiltonian, with only the z component of the DMI and a static field also parallel to z. With this choice, although different multiplets are mixed by the DMI (and hence transitions between them are allowed to first order), no anti-crossings are present in the level diagram of Fig. S1-(b). The effect of such a DMI is also a re-normalization of the energy gaps between different multiplets, evident also in zero field. We stress that a different form of the DMI (including also x or y components) would modify the energies and the inter-multiplet matrix elements without altering our conclusions.

We finally note that addressing the energy gaps between the lowest multiplets in Fig. S1-

(b) by EPR requires frequencies in the W-band range. This choice was motivated by the need to resolve all the different energy gaps. However, it is important to stress that proper pulse-shaping techniques could be employed to reduce leakage even in presence of closer energy levels, obtained for instance by an overall reduction of all the couplings in the spin Hamiltonian.

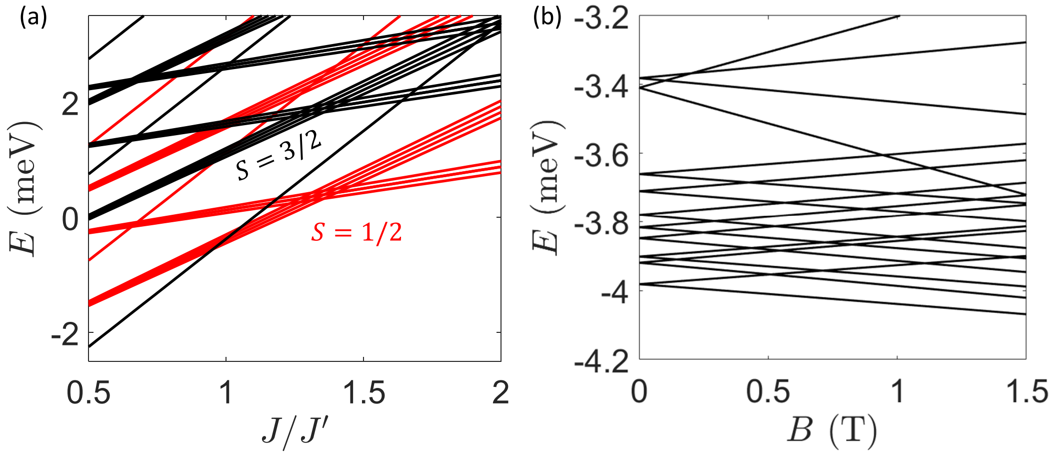

Figure S1: (a) Energy of the exchange multiplets with total spin $S = 1/2$ (red) and $S = 3/2$ (black), as a function of the ratio between the intra-triangular and the triangles-centre isotropic exchange interaction. To remove degeneracies we have here assumed $J_{1,3} = J_{2,3} = 1.05\ J$, $J_{4,6} = J_{5,6} = 0.95\ J$, $J_{4,5} = J_{1,2} = J$, $J_{1,7} = J_{2,7} = J_{3,7} = J_{4,7} = J_{5,7} = J_{6,7} = J'$. (b) Zeeman diagram of the full Hamiltonian (1) of the main text, as a function of the magnetic field, showing 8 doublets at low energy below the first $S = 3/2$ multiplet.

Derivation of the master equation

In this section, we derive the master equation used to describe decoherence given by the nuclear spin bath. The full Hamiltonian of the system and the bath can be written in the form $H = H_S + H_{SB} + H_B$, where H_S and H_B are the free Hamiltonians of the system and the bath, respectively, whereas H_{SB} describes their interaction. The Hamiltonian H_{SB} has the form

$$H_{SB} = \sum_{j=1}^{N_i} \sum_{n=1}^{N_s} \sum_{\substack{\alpha\beta= \\ x,y,z}} d_{jn}^{\alpha\beta} s_j^{\alpha} \otimes I_n^{\beta}. \tag{S1}$$

where $N_i = 7$ is the number of ions and N_s the number of nuclear spins in the bath. The operators s_j^α and I_n^α (with $\alpha = x, y, z$) are spin operators for the ions and the nuclear spins, respectively. The dipolar coefficients $d_{jn}^{\alpha\beta}$ are given explicitly in the main text. In the basis of the eigenstates $|\mu\rangle$ of the system Hamiltonian, defined by $H_S = \sum_\mu E_\mu |\mu\rangle\langle\mu|$, the interaction Hamiltonian in interaction picture, $H_{SB}(t) = e^{i(H_S+H_B)t} H_{SB} e^{-i(H_S+H_B)t}$, reads

$$H_{SB}(t) = \sum_{n=1}^{N_s} \sum_{\mu,\mu'} \sum_{\substack{\alpha\beta= \\ x,y,z}} \tilde{d}_{n,\mu\mu'}^{\alpha\beta} e^{i(E_\mu - E_{\mu'})t} L_{\mu\mu'} \otimes I_n^\beta(t). \tag{S2}$$

In this expression, we have denoted with $I_n^\beta(t) = e^{iH_B t} I_n^\beta e^{-iH_B t}$ the interaction picture coupling operators for the nuclear spins, we have used the notation $L_{\mu\mu'} = |\mu\rangle\langle\mu'|$, and the coupling coefficients read

$$\tilde{d}_{n,\mu\mu'}^{\alpha\beta} = \sum_{j=1}^{N_i} d_{jn}^{\alpha\beta} \langle\mu| s_j^z |\mu'\rangle . \tag{S3}$$

In the following, we consider only diagonal operators on the system ($\alpha = z$ in Eq. S1-S3), because transitions between different eigenstates of the system induced by the bath are forbidden by the large mismatch between system ($E_\mu - E_{\mu'}$ with $\mu \neq \mu'$) and bath energy gaps, compared to the couplings $d_{jn}^{\alpha\beta}$. Hence, terms $\propto L_{\mu\mu'}$ in Eq. (S2) can be neglected in the secular approximation yielding

$$H_{SB}(t) = \sum_{n=1}^{N_s} \sum_{\mu,\mu'} \sum_{\beta=x,y,z} \tilde{d}_{n,\mu\mu}^{\beta} L_{\mu\mu} \otimes I_n^\beta(t) \ . \tag{S4}$$

Note thus that the structure of the system eigenstates will be contained in $\tilde{d}_{n,\mu\mu'}^{\beta} \equiv \tilde{d}_{n,\mu\mu'}^{z\beta}$ through the matrix elements of the spin operators s_j^z. In interaction picture and under Born and Markov approximations, the reduced dynamics of the system can be described by the Markovian quantum master equation[S1]

$$\frac{d\rho(t)}{dt} = -\int_0^\infty dt' \mathrm{tr}_B[H_{SB}(t), [H_{SB}(t-t'), \rho(t) \otimes \rho_B]]. \tag{S5}$$

Here, $\rho(t)$ is the reduced density matrix of the system, obtained from the full system-bath density matrix $\rho_{SB}(t)$ by tracing over the bath degrees of freedom, $\rho(t) = \mathrm{tr}_B[\rho_{SB}(t)]$. Inserting Eq. (S4) into (S5) and using the shortcut notation $E_{\mu\mu'} = E_\mu - E_{\mu'}$, one arrives at the expression

$$\frac{d\rho(t)}{dt} = -\sum_{n,n'=1}^{N_s}\sum_{\mu,\nu}\sum_{\beta\beta'}\Big\{\tilde{d}^{\beta}_{n,\mu\mu}\tilde{d}^{\beta',*}_{n',\nu\nu}\tilde{\zeta}^{\beta\beta'}_{nn'}(0)\Big[L_{\mu\mu}L_{\nu\nu}\rho_S(t) \tag{S6}$$

$$- L_{\nu\nu}\rho_S(t)L_{\mu\mu}\Big] + \mathrm{h.c.}\Big\}, \tag{S7}$$

where we have also introduced the bath noise spectra

$$\tilde{\zeta}^{\beta\beta'}_{nn'}(\omega) = \int_0^\infty dt' G^{\beta\beta'}_{nn'}(t')e^{i\omega t}, \tag{S8}$$

as the (one-sided) Fourier transform of the bath correlation functions

$$G^{\beta\beta'}_{nn'}(t') = \mathrm{tr}_B\big[I^\beta_n(t)I^{\beta'\dagger}_{n'}(t-t')\rho_B\big] = \mathrm{tr}_B\big[I^\beta_n(t')I^{\beta'\dagger}_{n'}(0)\rho_B\big]. \tag{S9}$$

The correlation functions carry the information about the structure of the bath. In general, they are not accessible for interacting spin baths and can at best be approximated with numerical methods.[S2] Introducing the rates

$$\Gamma_{\mu\nu} = \frac{1}{2}\sum_{n,n'=1}^{N_s}\sum_{\beta,\beta'=x,y,z}\tilde{d}^{\beta}_{n,\mu\mu}\tilde{d}^{\beta',*}_{n',\nu\nu}\int_{-\infty}^{\infty} dt\ G^{\beta\beta'}_{nn'}(t)\ , \tag{S10}$$

$$S_\mu = \frac{1}{2i}\sum_{n,n'=1}^{N_s}\sum_{\beta,\beta'=x,y,z}\tilde{d}^{\beta}_{n,\mu\mu}\tilde{d}^{\beta',*}_{n',\nu\nu}\int_0^\infty dt\ [G^{\beta\beta'}_{nn'}(t) - G^{\beta\beta'}_{nn'}(-t)]\ , \tag{S11}$$

equation (S6) can then be brought into the form

$$\frac{d\rho(t)}{dt} = -i[H_{LS}, \rho(t)] + \sum_{\mu,\nu}\Gamma_{\mu\nu}[2L_{\nu\nu}\rho(t)L_{\mu\mu} - L_{\mu\mu}L_{\nu\nu}\rho(t) - \rho(t)L_{\mu\mu}L_{\nu\nu}], \tag{S12}$$

where $\mathcal{D}(L)\rho$ is a Lindblad dissipator,[S1] $\mathcal{D}(L)\rho = 2L\rho L^\dagger - L^\dagger L\rho - \rho L L^\dagger$. The term in the second line of Eq. (S12) is apparently not in Lindblad form, but it can be brought in such a form by diagonalizing the matrix $\Gamma_{\mu\nu}$.[S1] The Hamiltonian $H_{LS} = \sum_\mu S_\mu |\mu\rangle\langle\mu|$ is diagonal in the energy basis and only describes weak energy shifts of the system eigenstates. It will thus be neglected in the numerical simulations. The master equation (S12) will be used (with additional Hamiltonian terms describing the resonant pulses) in the numerical simulations. While diagonal elements of $\rho_S(t)$ are preserved, off-diagonal elements in the energy basis decay according to

$$\frac{d\rho_{\mu\nu}(t)}{dt} = (2\Gamma_{\mu\nu} - \Gamma_{\mu\mu} - \Gamma_{\nu\nu})\rho_{\mu\nu}(t), \tag{S13}$$

with

$$\Gamma_{\mu\nu} = \sum_{j,j'=1}^{N_i} \sum_{\alpha,\alpha'=x,y,z} C^{\alpha\alpha'}_{jj'} \langle\mu| s^\alpha_j |\mu\rangle \langle\nu| s^{\alpha'}_{j'} |\nu\rangle \ , \tag{S14}$$

as given in the main text. In the last line, Eq. (S14), we have introduced

$$C^{\alpha\alpha'}_{jj'} = \sum_{n,n'=1}^{N_s} \sum_{\beta,\beta'=x,y,z} \zeta^{(\beta\beta')}_{nn'}(0) d^{\alpha\beta}_{jn} d^{\alpha'\beta',*}_{j'n'}, \tag{S15}$$

with $\zeta^{(\beta\beta')}_{nn'}(0) = \int_{-\infty}^{\infty} dt\ G^{\beta\beta'}_{nn'}(t)$. Solving (S13) thus gives the exponential decay $\rho_{\mu\nu}(t) = e^{-\gamma_{\mu\nu}t}\rho_{\mu\nu}(0)$ for the off-diagonal elements, with decay rate $\gamma_{\mu\nu} = -2\Gamma_{\mu\nu} + \Gamma_{\mu\mu} + \Gamma_{\nu\nu}$. In the following, since our focus in on how the structure of system eigenstates, rather than the structure of the bath, impacts decoherence, we will assume that the bath noise spectrum is constant at zero energy, $\zeta^{\beta\beta'}_{nn'}(0) = \zeta_0$. We have checked, by considering a random distribution of $\zeta^{\beta\beta'}_{nn'}(0)$, that this choice does alter our conclusions.

Comparison between F and C cases

In order to compare the effect of ferromagnetic (**F**) vs competing (**C**) exchange interactions, we consider the molecular structure reported in Fig. 1-(a) of the main text with either

negative (**F**) or positive (**C**) $J_{i,j}$. For **F**, one can easily compute the elements of $\Gamma_{mm'}$ within the ground $S = 9/2$ multiplet:

$$\Gamma^{\mathbf{F}}_{mm'} = \zeta_0 \sum_{jj'} \sum_{\alpha\alpha'} c^{\alpha\alpha'}_{jj'} \langle m|s^{\alpha}_j|m\rangle \langle m'|s^{\alpha'}_{j'}|m'\rangle \tag{S16}$$

$$= \zeta_0 \sum_{jj'} \sum_{\alpha\alpha'} c^{\alpha\alpha'}_{jj'} p_j p_{j'} \langle m|S^{\alpha}|m\rangle \langle m'|S^{\alpha'}|m'\rangle \tag{S17}$$

$$= \zeta_0 \left(\sum_{jj'} c^{zz}_{jj'} p_j p_{j'} \right) mm' = \frac{mm'}{T_2}. \tag{S18}$$

Here $c^{\alpha\alpha'}_{jj'} = \sum_{nn'} \sum_{\beta\beta'} d^{\alpha\beta}_{jn} d^{\alpha'\beta'}_{j'n'}$ includes the effect of the coupling between system and bath spins. By assuming isotropic g_j, one gets $d^{\alpha\beta}_{jn} = g_j g_N \mu_B \mu_N (3 r^{\alpha}_{jn} r^{\beta}_{jn}/|r_{jn}|^2 - \delta_{\alpha\beta})/|r_{jn}|^3$, g_N is the hydrogen nuclear spin spectroscopic factor, μ_N is the nuclear magneton and r^{α}_{jn} is the α component of the distance between the j-th electronic spin and the n-th nuclear spin. ζ_0 is the bath spectral function, assumed to be a constant.

Here we have exploited the Wigner-Eckart theorem within the S multiplet $\langle m|s^z_j|m\rangle = p_j \langle m|S^z|m\rangle$. Then, neglecting small anisotropy effects, $\langle m|S^z|m\rangle = m$. We have introduced here a phenomonological coherence time (as usually done in experiments), defined as $T_2 = \left(\zeta_0 \sum_{jj'} c^{zz}_{jj'} p_j p_{j'}\right)^{-1}$. This can be substituted for comparison in the analogous expression for the antiferromagnetic case, thus getting rid of the unknown bath spectral function ζ_0.

$$\Gamma^{\mathbf{C}}_{\mu\nu} = \zeta_0 \sum_{jj'} \sum_{\alpha\alpha'} c^{\alpha\alpha'}_{jj'} \langle \mu|s^{\alpha}_j|\mu\rangle \langle \nu|s^{\alpha'}_{j'}|\nu\rangle \ , \tag{S19}$$

$$= \zeta_0 \sum_{jj'} c^{zz}_{jj'} \langle \mu|s^{z}_j|\mu\rangle \langle \nu|s^{z}_{j'}|\nu\rangle \ , \tag{S20}$$

$$= \frac{1}{T_2 \left(\sum_{jj'} c^{zz}_{jj'} p_j p_{j'} \right)} \sum_{jj'} c^{zz}_{jj'} \langle \mu|s^{z}_j|\mu\rangle \langle \nu|s^{z}_{j'}|\nu\rangle \ . \tag{S21}$$

Impact of the structure of the eigenstates on decoherence

In the following, we argue that the essential dependence on the system eigenstates that rules decoherence at short times, as outlined above, is confirmed also when avoiding to make Markov approximations for the bath. In particular, by restarting from the microscopic system-bath interaction Eq. (S1) and neglecting off-diagonal couplings (which is justified since the gaps in the system are much larger than the coupling to the bath), the interaction-picture system-bath Hamiltonian in the basis of system eigenstates $|\mu\rangle$ can be written in the form $H_{SB}(t) = \sum_\mu |\mu\rangle\langle\mu| \otimes H_\mu(t)$ with

$$H_\mu(t) = \sum_n h_n^\mu I_n^z(t) \ , \tag{S22}$$

and coefficients $h_n^\mu = \sum_j d_{jn}^{zz} \langle\mu| s_j^z |\mu\rangle$. We will indicate with $U_\mu^\pm(t)$ the propagator generated by $\pm H_\mu(t)$. Although $U_\mu^\pm(t)$ do have the form of a simple exponential of $\pm H_\mu(t)$ because of the time dependence, for short times (compared to the inverse of the system-bath couplings) it can be estimated with a Magnus expansion,

$$U_\mu^\pm(t) = \exp\left(\mp i \int_0^t dt \ H_\mu(t) - \frac{1}{2}\int_0^t dt_1 \int_0^{t_1} dt_2 \ [H_\mu(t_1), H_\mu(t_2)] + \dots\right) \ , \tag{S23}$$

$$\simeq \exp\left(\mp i \sum_n h_n^\mu \int_0^t dt_1 \ I_n^z(t_1) - \frac{1}{2}\sum_{n,n'} h_n^\mu h_{n'}^\mu \int_0^t dt_1 \int_0^{t_1} dt_2 \ [I_n^z(t_1), I_{n'}^z(t_2)] + \dots\right) \ , \tag{S24}$$

$$(U_\mu^\pm(t))^\dagger \simeq \exp\left(\pm i \sum_n h_n^\mu \int_0^t dt_1 \ I_n^z(t_1) + \frac{1}{2}\sum_{n,n'} h_n^\mu h_{n'}^\mu \int_0^t dt_1 \int_0^{t_1} dt_2 \ [I_n^z(t_1), I_{n'}^z(t_2)] + \dots\right) \ , \tag{S25}$$

where we have used Eq. (S22) and kept terms up to second-order in system-bath coupling. The free evolution of the system coherences (in interaction picture for the system) can then be written like

$$\rho_{\mu\nu}(t) = \mathrm{tr}_B\left[U_\mu^+(t) \langle\mu| \rho_{SB}(0) |\nu\rangle (U_\nu^+(t))^\dagger\right] \ , \tag{S26}$$

where ρ_{SB} is the system-bath density matrix. Under Born approximation for the initial state, $\rho_{SB}(0) = \rho(0) \otimes \rho_B(0)$, this gives the coherence decay $\rho_{\mu\nu}(t) = \mathcal{L}_{\mu\nu}(t)\rho_{\mu\nu}(0)$ with

$$\mathcal{L}_{\mu\nu}(t) = \mathrm{tr}_B\left[U_\mu^+(t)\rho_B(0)(U_\nu^+(t))^\dagger\right] . \tag{S27}$$

Assuming that the time t is small as compared to the energy scales in $H_{B,\pm}^{(\mu)}$, we can use Baker-Campbell-Hausdorf formula, $e^{-iAt}e^{-iBt} = e^{-i(A+B)t-\frac{t^2}{2}[A,B]+O(t^3)}$, together with the Magnus expansion (S23) to approximate the product of exponentials. Equation (S27) then gives, for $\mu \neq \nu$,

$$\mathcal{L}_{\mu\nu}(t) \simeq \mathrm{tr}_B\Big[\exp\Big(i\sum_n (h_n^\nu - h_n^\mu)\int_0^t dt_1 I_n^z(t_1) \tag{S28}$$

$$+\frac{1}{2}\sum_{n,n'}(h_n^\nu h_{n'}^\nu - h_n^\mu h_{n'}^\mu)\int_0^t dt_1 \int_0^{t_1} dt_2 [I_n^z(t_1), I_{n'}^z(t_2)] \tag{S29}$$

$$+\frac{1}{2}\sum_{n\neq n'} h_n^\nu h_{n'}^\mu \int_0^t dt_1 \int_0^t dt_2 [I_n^z(t_1), I_{n'}^z(t_2)]\Big)\rho_B(0)\Big]. \tag{S30}$$

Keeping only the leading term up to first order in t gives $\mathcal{L}_{\mu\nu}(t) = \prod_n \cos[(h_n^\nu - h_n^\mu)t/2]$. This is well approximated at short times by the Gaussian decay $\mathcal{L}_{\mu\nu}(t) \approx e^{-\Gamma t^2}$, with

$$\Gamma = \sum_n (h_n^\nu - h_n^\mu)^2/4. \tag{S31}$$

Hence, even without the Markov approximation, the short-time decay rate of the system coherences is related to the spin structure of the systems by a squared sum of weighted differences between expectation values of local spin operators.

Process tomography and derivation of the code words

We consider a subset of the system Hilbert space of dimension d. Starting from the solution of the Lindblad equation for the pure dephasing mechanism described in this work,

$$\rho(t) = \sum_{mm'} \rho_{mm'}(0) e^{-\gamma_{mm'} t} |m\rangle \langle m'| , \tag{S32}$$

we look for a set of operators $\{E_k\}$ (such that $\sum_k E_k E_k^\dagger = I$) providing a Kraus decomposition for the system density matrix at a given time t at which the error correction will be performed, i.e.

$$\rho(t) = \sum_k E_k \rho(0) E_k^\dagger. \tag{S33}$$

The operators E_k represent the different quantum errors occurring on the system. From Eq.(S32) we note that the E_k operators must be diagonal. Hence, only d linearly independent E_k exist. These can in general be determined through quantum process tomography, as described in Ref.[S3] (Sec. 8.4.2), which is simplified here given the diagonal structure of the E_k. We first decompose the E_k on a basis of diagonal operators $\tilde{E}_i$,

$$E_k = \sum_{i=1}^{d} \epsilon_{ik} \tilde{E}_i \tag{S34}$$

and we re-write Eq. (S33) as

$$\rho(t) = \sum_{i,j=1}^{d} \chi_{ij} \tilde{E}_i \rho(0) \tilde{E}_j^\dagger, \tag{S35}$$

with $\chi_{ij} = \sum_k \epsilon_{ik} \epsilon_{jk}^*$. By choosing the operators $\{\tilde{E}_i\}$ as projectors onto the eigenstates $\tilde{E}_\mu = |\mu\rangle\langle\mu|$ and comparing with Eq. (S32) one easily finds $\chi_{ij} = e^{-\gamma_{ij} t}$.

Bi diagonalizing χ, one then finds the Kraus operators. Indeed, being $V^\dagger$ the matrix that diagonalizes χ and ξ the vector of the eigenvalues, we can rewrite

$$\chi_{ij} = \sum_m V_{im} \xi_m V_{jm}^*, \tag{S36}$$

from which one obtains

$$E_k = \sqrt{\xi_k} \sum_\mu V_{\mu k} \tilde{E}_\mu = \sqrt{\xi_k} \sum_\mu V_{\mu k} \left|\mu\right\rangle\!\left\langle\mu\right| . \tag{S37}$$

Detection of a number n of different errors is possible provided the Hilbert space has at least dimension $2n$: indeed, one needs at least $2n$ orthogonal states for mapping the effect on each error on the two logical states, $E_k \left|0_L\right\rangle$ and $E_k \left|1_L\right\rangle$, to distinguishable states. In the systems considered here, one cannot in general correct all d operators E_k, but only a subset of size $d/2$. It is thus important to identify the error operators that give largest contribution to reconstruct $\rho(t)$. To this end, we observe that the squared norm $\mathrm{tr}(E_k^\dagger E_k)$ of E_k is proportional to the eigenvalues ξ_k of χ. Hence, by sorting the eigenvalues of the χ matrix in decreasing order, the required hierarchy for the Kraus operators E_k is obtained. Once the dominant error operators E_k are determined, a quantum error correction scheme is formulated by finding logical states $\left|0\right\rangle_L$ and $\left|1\right\rangle_L$ fulfilling Knill-Laflamme conditions (KLc), Eq. 4 of the main text.

To this end, we consider code words defined on orthogonal superpositions of the the system eigenstates:

$$\left|0_L\right\rangle = \sum_{\mu \in A} a_\mu \left|\mu\right\rangle \tag{S38}$$

$$\left|1_L\right\rangle = \sum_{\mu \in B} b_\mu \left|\mu\right\rangle , \tag{S39}$$

where A and B are disjoint subsets of $d/2$ levels. With this choice, $\left|0_L\right\rangle$ and $\left|1_L\right\rangle$ are not mixed by diagonal E_k operators by construction, thus automatically fulfilling the second KLc,[S4] i.e. $\left\langle 0_L\right| E_k E_j^\dagger \left|1_L\right\rangle = 0$. Exploiting the decomposition of E_k into projectors on the eigenstates (Eq. S37), the first KLc $\left\langle 0_L\right| E_k E_j^\dagger \left|0_L\right\rangle = \left\langle 1_L\right| E_k E_j^\dagger \left|1_L\right\rangle$ can be re-written as

$$\sqrt{\xi_k \xi_j} \sum_{\mu \in A} V_{\mu k} V_{\mu j}^* |a_\mu|^2 = \sqrt{\xi_k \xi_j} \sum_{\mu' \in B} V_{\mu' k} V_{\mu' j}^* |b_{\mu'}|^2 . \tag{S40}$$

This set of conditions for $j, k = 0, ..., d/2 - 1$, together with the normalisation conditions $\sum_\mu |a_\mu|^2 = \sum_\mu |b_\mu|^2 = 1$, form a linear system of equations in the variables $|a_\mu|^2$, $|b_\mu|^2$. This system is in general overdetermined, but numerically underdetermined in practical cases. Hence, at least one solution can be found, either by matrix pseudo-inversion or by numerical optimisation.

Actual implementation of Quantum Error Correction

To identify the different errors E_k we generalise the procedure usually exploited in qubit-based quantum error correction codes to measure a set of stabilizers.[S5]

Error detection

The scheme we follow is summarised in Fig. 3(a) of the main text. Here we detail each step and follow the corresponding evolution of system state. To do this, we first introduce an orthonormal basis set of so-called *error words* $\{|e_k^0\rangle, |e_k^1\rangle\}$, $k = 0, ...d/2 - 1$ spanning the *error spaces* defined as $\text{span}\{E_k|0_L\rangle\}_{k=1,...,d/2}$ and $\text{span}\{E_k|1_L\rangle\}_{k=1,...,d/2}$, respectively. For performing the error detection, we need to realize a measurement distinguishing different error words, described by projectors $P_k = |e_k^0\rangle\langle e_k^0| + |e_k^1\rangle\langle e_k^1|$. In this way, the system is projected post-measurement into a known error word in both error spaces, without collapsing the stored information. This then allows one to implement a corresponding k-dependent recovery operation. Implementing these projectors onto superposition states of the system eigenstates directly can be hard experimentally. Therefore, in our scheme we first perform a transformation that entangles each of the $d/2$ error words $\alpha |e_k^0\rangle + \beta |e_k^1\rangle$ with a different eigenstate of an ancillary $d/2$-level qudit. The desired projectors on the system are then obtained by measuring the ancilla in its eigenbasis.

The sequence of operations (see Fig. 3 of the main text) is the following:

0. Start with the qudit in a generic logical state $|\psi\rangle$ (on which pure dephasing has possibly

occurred) and a $d/2-$levels ancillary qudit in its ground state $|0\rangle$, i.e. the combined ancilla-qudit state is the tensor product $|\Phi\rangle = |0\rangle \otimes |\psi\rangle$.

1. Apply a qudit Hadamard gate H$= \sqrt{\frac{2}{d}} \sum_{j,k=0}^{d/2-1} e^{i\frac{4\pi}{d}jk} |j\rangle \langle k|$ on the $d/2$ levels ancilla, which brings its state to the uniform superposition $|+\rangle = \sqrt{\frac{2}{d}} \sum_k |k\rangle$. After this transformation, the qudit-ancilla state is $|\Phi\rangle = |+\rangle \otimes |\psi\rangle$.

2. Implement a conditional evolution of the system, depending on the state of the ancilla. In particular, for each state of the ancilla, a different unitary operator is applied, $U_k = \sum_{j=0}^{d/2-1} \sum_{\ell=0,1} e^{i\frac{4\pi}{d}jk} |e_j^\ell\rangle \langle e_j^\ell|$. The resulting state can be understood by expanding $|\psi\rangle$ as a superposition of the error words, namely $|\psi\rangle = \sum_j \sum_\ell \eta_j^\ell |e_j^\ell\rangle$. By simple algebra one then finds $|\Phi\rangle = \sqrt{\frac{2}{d}} \sum_{kj} \sum_\ell e^{i\frac{4\pi}{d}jk} \eta_j^\ell |k\rangle |e_j^\ell\rangle$.

3. Apply to the ancilla $\mathrm{H}^\dagger = \sqrt{\frac{2}{d}} \sum_{jk} e^{-i\frac{4\pi}{d}jk} |k\rangle \langle j|$, ending up with $\mathrm{H}^\dagger |\Phi\rangle = \sum_j \sum_\ell \eta_j^\ell |j\rangle |e_j^\ell\rangle$, after exploiting $\frac{2}{d} \sum_k e^{i\frac{4\pi}{d}(j-j')k} = \delta_{jj'}$. This is an entangled ancilla-qudit state in which each component on the ancilla is in one-to-one correspondence with a specific error word.

4. Measure the state of the ancilla, thus collapsing the qudit into a given error word. Note that the superposition of $|e_k^0\rangle$ and $|e_k^1\rangle$ is the same as was at the beginning of the sequence, i.e. the quantum information stored into the coefficients α, β of the initial state $\alpha |0_L\rangle + \beta |1_L\rangle \rightarrow \alpha |e_k^0\rangle + \beta |e_k^1\rangle$ is preserved.

5. Having identified an error word, apply the corresponding recovery operation $R_k = \sum_\ell |e_0^\ell\rangle \langle e_k^\ell| +$h.c. and then rotate back $\alpha |e_0^0\rangle + \beta |e_0^1\rangle \rightarrow \alpha |0_L\rangle + \beta |1_L\rangle$.

Implementation of $\mathbf{U}_k$

Each of the unitaries U_k can be implemented by a quantum simulation of the associated effective Hamiltonian $\tilde{H}_k = -\sum_{j,\ell} \frac{4\pi}{d} jk |e_k^\ell\rangle \langle e_k^\ell|$ which generates U_k, i.e. $e^{-i\tilde{H}_k} = \mathrm{U}_k$. The whole quantum simulation can be implemented via two sets of simultaneous pulses for the

diagonal and off-diagonal terms of $\tilde{H}_k$ by a Suzuki-Trotter (ST) decomposition in which all off-diagonal terms $\langle m|\tilde{H}_k|m'\rangle$ of $\tilde{H}$ are implemented in parallel via a set of pulses resonant with $|m\rangle \rightarrow |m'\rangle$ transitions. The non-commuting diagonal terms $\langle m|\tilde{H}_k|m\rangle$ are instead simulated by semi-resonant excitations of a state of the ancilla, conditioned by each $|m\rangle$ state.

Remarkably, the length of the sequence does not depend on the number of steps N in the ST decomposition. Indeed, each of the aforementioned rotations has a duration proportional to the rotation angle, which is fixed if one performs a sequence of N rotations of θ/N. Hence, the error of the Suzuki-Trotter decomposition does not represent a limitation of the current approach, because it can be made arbitrarily small by increasing N, without altering the actual duration of the sequence.

Quantum gates between encoded states

We illustrate our scheme to implement $R_x(\vartheta)$ gates between encoded states for $d = 4$, but the procedure can be easily extended to a larger number of levels encoding the logical state. In this case, $|0_L\rangle$ and $|1_L\rangle$ are real superposition of two eigenstates each, namely $|0_L\rangle = \cos\phi\,|1\rangle + \sin\phi\,|4\rangle$ and $|1_L\rangle = \cos\chi\,|2\rangle + \sin\chi\,|3\rangle$, where the eigenstates are labeled as $|\mu\rangle$, $\mu = 1, ..., 4$.

We define a logical $R_x(\vartheta)$ as

$$\begin{aligned} R_x(\vartheta) &= \cos\frac{\vartheta}{2}\left(|0_L\rangle\langle 0_L| + |1_L\rangle\langle 1_L| + |\varepsilon_0\rangle\langle\varepsilon_0| + |\varepsilon_1\rangle\langle\varepsilon_1|\right) \\ &- i\,\sin\frac{\vartheta}{2}\left(|0_L\rangle\langle 1_L| + |1_L\rangle\langle 0_L| + |\varepsilon_0\rangle\langle\varepsilon_1| + |\varepsilon_1\rangle\langle\varepsilon_0|\right), \end{aligned} \tag{S41}$$

where we have introduced the additional states $|\varepsilon_0\rangle = \sin\phi\,|1\rangle - \cos\phi\,|4\rangle$ and $|\varepsilon_1\rangle = \sin\chi\,|2\rangle - \cos\chi\,|3\rangle$ to complete unitarity. This transformation is generated by the effective Hamiltonian

$$\mathcal{H} = |0_L\rangle\langle 1_L| + |1_L\rangle\langle 0_L| + |\varepsilon_0\rangle\langle\varepsilon_1| + |\varepsilon_1\rangle\langle\varepsilon_0|, \tag{S42}$$

meaning that $R_x(\vartheta) = e^{-i\mathcal{H}\vartheta/2}$. In the basis of the eigenstates of the system, $\mathcal{H}$ assumes the form

$$\mathcal{H} = \cos(\phi - \chi)\left(|1\rangle\langle 2| + |3\rangle\langle 4|\right) + \sin(\phi - \chi)\left(-|1\rangle\langle 3| + |2\rangle\langle 4|\right) + \text{h.c.}, \tag{S43}$$

Hence, a generic $R_x(\vartheta)$ rotation can be obtained by four simultaneous pulses of angles $2\arcsin\left[\cos(\phi-\chi)\sin\frac{\vartheta}{2}\right]$ (resonant with $|1\rangle \to |2\rangle$ and $|4\rangle \to |3\rangle$ transitions) and $2\arcsin\left[\pm\sin(\phi-\chi)\sin\frac{\vartheta}{2}\right]$ (resonant with $|1\rangle \to |3\rangle$ and $|2\rangle \to |4\rangle$ transitions). In the simulations reported in the main text, we have $\phi \approx \chi$ and hence only two ϑ pulses resonant with $|1\rangle \to |2\rangle$ and $|4\rangle \to |3\rangle$ transitions are needed.

References

(S1) Breuer, H.-P.; Petruccione, F. *The theory of open quantum systems*; Oxford University Press (Oxford), 2010.

(S2) Petiziol, F.; Chiesa, A.; Wimberger, S.; Santini, P.; Carretta, S. Counteracting Dephasing in Molecular Nanomagnets by Optimized Qudit Encodings. *npj Quantum Inf.* **2021**, *7*, 133.

(S3) Nielsen, M. A.; Chuang, I. L. *Quantum Computation and Quantum Information: 10th Anniversary Edition*; Cambridge University Press: Cambridge, 2011.

(S4) Knill, E.; Laflamme, R. Theory of Quantum Error-Correcting Codes. *Phys. Rev. A* **1997**, *55*, 900–911.

(S5) Devitt, S. J.; Munro, W. J.; Nemoto, K. Quantum Error Correction for Beginners. *Rep. Progr. Phys.* **2013**, *76*, 076001.
